# Opium: Its Influence in Febrile, Inflammatory, and Other Diseases

**Published:** 1860-01

**Authors:** A. S. Hudson

**Affiliations:** Prof. of Physiology, Pathology, and Clinical Medicine in Rush Medical College


### ARTICLE 5.

### OPIUM:

### ITS INFLUENCE IN FEBRILE, INFLAMMATORY, AND OTHER DISEASES.

A PRIZE ESSAY. BY A. S. HUDSON, M. D.,

Prof. of Physiology, Pathology, and Clinical Medicine in Rush Medical College.

*Editors of Chicago Medical Journal:*

GENTS: An essay of mine, under the above caption, having been honored with acceptance and publication by the State Medical Society, appears in the transactions of that Society, imperfectly printed. Not being in the city at the time, I could not read or correct the proof. If in your journal you can spare the room, I would be glad to have it re-printed in a more intelligible form.

Yours, respectfully,

A. S. HUDSON.

—

Give anguish a tongue, and reason to a remedy,
And they will commune together in blissful concord.

To calculate the worth of an agent by the same rule from which Napoleon estimated the worth of a man, namely, by what he can do,—it must conceded that opium is the giant of the materia medica. It is one of the oldest remedies of the catalogue. Among the changes of professional fashion, or of medical caprice, none has maintained the perpetuity of a large respect equal to opium.

It has many friends, but few enemies. It has many capabilities, but few defects. It has mighty powers, yet its mischiefs, through error and depravity of usage, are among the list of minimums.

Ethereal theories in medicine, and extravagant systems of practice, have, for the last few centuries, arisen every fifty years; opium finds a ready niche in the newly created temple of each, and quietly lends, as ever, its faithful charms to the suffering world. Opinions change, and doctrines pass away, but opium is still respected as lord of physical woe, and held ready for the oft arising pang of sharp emergency.

In discussing the therapeutics of opium, it may be systematic to divide the subject under three natural heads.

1. The physiological action of opium.
2. The general therapeutics of opium.
3. The powers of opium in febrile and inflammatory diseases.

1. *The Physiological action.*—Opium is one of those agents whose energy is not often obliterated by mental action, nor augmented by the coadjutive unction of faith. Its medical properties depend wholly upon the inherent potencies of itself. Morbid action and intensified mentality may modify its forces, but they do not obliterate them.

It acts with the same fidelity upon the unconscious infant, as in the vigor of manhood writhing under throes of pain, or in the time-furrowed system of the aged and demented.

In minor-sized doses, opium is defined as a stimulant, augmenting the motion of the blood, the mental faculties, the nervous tone, and induces a feeling of muscular comfort.

In a larger dose, the pulse is lessened in number, but its volume is increased, respiration diminished. The stomach frowns upon, and objects to its natural duties, and often acts in reversion by emesis. A dull and lazy pain attends. The pain in the stomach is a sort of constriction or corrugation, by which its walls seem to be drawn together, or strongly compressed. The tongue and fauces are dry, and there is a cutaneous pruritus. The mental faculties enjoy a serene pitch of self-entertainment, or a prepossessing degree of repose. Sorrow has fled, and the twinge of pain is wrapped in the magic spell of physical felicity, never again to return, or to return with diminished consequence.

The third degree of operation is a toxic influence. It paralyzes the respiratory muscles, respiration is labored and even lost. There is stupor, and contracted pupil, livid countenance, and muscular relaxation; giddiness.

Constipation is an effect that follows any sized dose.

Pereira states that opium is an aphrodisiac, and augments the venereal propensity. So far as my own observation extends, in studying this effect upon myself and others, I must, in all deference to the great English author above-mentioned, enter the averment of a stout denial; and state to the contrary, that it invariably allays even natural salacity. This point is further sustained by the testimony of opium eaters, as the "Baltimore Bard," and a writer (a reclaimed subject) in the *N. Y. Medical Times*, for 1849. To this excellence of opium—the non-excitation of the sexual functions—we must attribute much of its curative ability in some inflammatory diseases we shall specify anon.

Opium is a cerebo-spinant, and acts, according to Flourens, on the cerebal lobes; while belladonna operates on the corpora quadrigemina. The kind of influence displayed over the mind by this drug, is by no means uniform. That blissful revery so highly enjoyed by the opium eaters, is an ecstasy which I am disposed to believe follows its use in only about half the cases; the other half will experience the hypnotic and anodyne effects, but no special mental elevation. There is, therefore, no danger of this latter half becoming intemperate with opium. Nor does the truth of the statement, that the habit of consuming the drug always requires the victim to increase the size of the dose from month to month to maintain the same feeling of supreme felicity, reach any further than perhaps half the cases. The individual mentioned in the *N. Y. Medical Times*, writes thus of himself: "After commencing or recommencing the use of opium, I always reached the full amount which as a *habit* I ever used, that is, either an half an ounce of opium or a quarter of an ounce of morphine in a week." Such also is the case with the only opium-eater with whom I have met. She has taken morphine for six years, and by a dose once or twice a day, she consumes about half a drachm in a week. She has recently abandoned the habit, by making a daily dimunition of the size of the dose.

There are many idiosyncrasies which rest opposed to the friendly influence of opium. Some patients, mostly females, are kept awake through its action, instead of enjoying the somnific repose it promises. This minor objection can be remedied in two ways. The coveted sleep, which stands off like a ghost at arms-length, comes at last, but at a late hour. It is belated, and may be hastened by giving a larger dose, or giving the same dose a few hours earlier. To increase the dose one-quarter or one-half the usual amount, may generally be done without prejudice to the case.

Retention of the urine, through seeming paralysis of the vesico-genital organs, is a frequent result of a full dose; it however, passes away in six to twelve hours.

The next and most abiding objection to opium, is nausea, which is a secondary effect of its operation. This is the most vexatious with female patients, and arises in from three to ten hours after its ingestion. Some will vomit and retch for many hours, while others again are only slightly sickened. As a general rule, individuals who use tobacco, are exempt from the effect of opium. The evil property is supposed to depend upon the narcotine it contains. Accordingly, Mitchell's Therapeutics gives the subjoined formula, which the author, with much scientific looseness of expression, states to be without narcotine. R Sixteen grains of pure morphia, eight grains of crystalized citric acid, are to be dissolved in an ounce of water, and just enough tincture of cochineal to give it a pink tinge. Dose, 5 to 25 drops.

He appends the random praise that "I have found it a very pleasant opiate." Query? What becomes of the narcotine here? Is it impotently neutralized? Is it expelled? He does not say; nor does he state that his preparation is free from narcotine and from nauseating qualities. Orfila at one time declared narcotine to be inert, but afterwards thought it acted like morphia, and had an operation remarkable and peculiar.

Bally asserts that in a solid state it is inert, for 129 grains given at a dose produced no obvious effect. Dr. Roots gave scruple doses without injury, but from its bitterness employed it in intermittents in place of quinine. The truth is, I believe narcotine possesses but little activity, and I presume the first experimentors with it, employed an impure article. (Pereira.)

2. *General Therapeutics of Opium.*—The principal property of opium, and the one for which it is mostly used by the majority of practitioners, is its anodyne or hypnotic characteristic. Without lisping a word tending to lessen the high estimate of this quality, yet, when contrasted with its other powers, it is, if any thing, the inferior. Opium is not merely a soothing comforter, beguiling turbulent agitation into reluctant sleep, and thus acting as a palliative, but it is an instrument of veritable cure. It not only abates the sting, but it removes the thorn; while it carries life's struggling vessel in tranquility over a heavy sea of disease, it will often, at the same time quell the tempest. Thus, in gastralgia, and in contorting colic, a large dose brings sovereign relief, and thwarts their return for many weeks or months, or perhaps never to return. But should they relapse, the same happy effects may be confided in again. And here let me say, for the treatment of these assassinlike maladies, that opium has no rival. We challenge the profession to exhibit its master, either for temporary or perpetual relief. Generally, from first to last, it is the remedy. Bleeding, warm bath, nauseants, may assist its action, but they are mere adjuncts and subordinates. These are among the cases in which the physician does not leave the patient till he is convalescing.

Your honorable Society virtually asks the question, "Is opium a febrifuge, or does it possess antiphlogistic properties?" The reply to this question will be considered in the last part of this dissertation. But let us first inquire, *Is opium an alterative?* Does it effect such a changed state of action as to bring it under the definition of an alterative? We claim it does; it is an alterative of no despisable energy. Mr. F. C. Skey (*London Lancet*, Feb., 1855,) makes the following remarks on the efficacy of opium in the treatment of chronic ulcers in the legs: "I venture to attribute to this remarkable drug, (opium) the property of promoting the formation of healthy granulations on a surface that, notwithstanding all the previous appliances of surgery, is yet flat, pale, and ungranulating. Now, there is no example of the power of opium to effect this more conclusive, or in which there is more work to be done, than that form of disease of which I am speaking—which consists of a gap formed on the surface of the body, of greater or less depth and diameter, and in which there exists not even a trace of curative action—and yet the object is accomplished by means of this agent, and often with remarkable celerity."

Mr. Skey beholds similar properties in this medicine in the treatment of senile gangrene, which is a painless disease. He says it maintains the balance of the circulation. "How much of meaning is attached to these words! How many affections of the bodily frame may not be brought within the range of this definition! Take the common chilblain; what is it but a local congestion of blood caused by defective capillary power? there is no better remedy than opium; cold feet, as characterizing a person or a constitution, is equally relieved; senile gangrene, the result of arrest of the capillary circulation, or its apparent opposite; local hyperænia; these diseases, one and all, manifest a loss of local power, a failure in the balance of the circulation. The influence of opium in such conditions is that of promoting a genial warmth over the system, a glow exactly resembling, in fact identical with, that produced by the re-action on the system which is caused by the cold bath. *It is local health*, and the sensation is most agreeable. * * * * * I have related to you the particulars of several cases of chronic ulcer in which recovery was attributable to the medical properties of opium, and almost to opium alone. The character of these ulcers strongly marks the inactivity of their nature, and hence the class of society to which they belong. They are marked by a flat base, which indicates by its pale, flabby uniformity of surface, that no reparatory action has approached it. It is often surrounded by a thick, high ridge of lymph, covered by unhealthy integuments. The depth of the ulcer, which may be seven or eight lines, is caused partly by the ridge, and partly by the excavation of the ulcer below the natural level of the healthy integuments. So long as this ridge exists, although granulations may form, and will form from the date of the employment of the opium, yet cicatrization will never complete the process of cure unless the wound or ridge be absorbed. Now the action of opium is not alone exhibited in the development of healthy granulations, but in the entire complement of such actions as are required by the sore, viz: the formation of new material and the absorption of the old."

We have other authorities on this head; statements which, like the above, by their conceded weight of authority, carry with them a breast of proof, which well supplies the place of facts, and shortens the line of argument. After ample opportunity to render his observations in syphilis authoritive, M. Rodet comes to the conclusion that it is an error to regard opium as a mere succedaneum of mercury; it acting, in fact, most efficaciously in just those cases in which mercury is of least use, and *vice versa*.

When in chancres there is a manifest tendency to phagedena, or they are irritable and painful, mercury should be rigidly forbidden, while opium is always useful in assuaging the pain, diminishing the irritability, and modifying the suppurative process. But the case in which truly remarkable effects are produced by opium, and in which it acts almost as a specific, is that of *phagedenic, serpiginous ulcers.*

M. Rodet, prior to resorting to large doses of opium, treated them by a great variety of means, without any satisfactory result. The cyanide of potassium, although incapable of healing them, effected considerable amendment in their appearance. The iodide of potassium was found to be of no avail; and those who believed in its efficacy have confounded those primary ulcers with other serpiginous ulcerations, which much resemble them in appearance, but which appear during the tertiary period of syphilis. As to mercury, not only is it useless, but mischievous, and should be rejected. When the ulcers decidedly manifest these appearances, opium exerts a most beneficial effect upon them. If some obstacle impedes the progress of cicatrization, the opium does not operate against this which has to be removed by other means; and the neglect of this precaution may lead to the loss of much time, and the taking of large unnecesary quantities of opium. The opium must not be given in too divided doses, as if the stomach be kept too constantly under its action, digestion is interfered with. The entire daily quantity should be taken at two doses, morning and evening, leaving a sufficient space of time after meals to secure the completion of the digestive process before administering it. If this point is not attended to, indigestion, accompanied with nausea, vomiting, diarrhœa, sweating, prostration, a cephalalgia, etc., comes on; compelling a temporary cessation of the remedy, and reacting unfavorably on the ulcer. Sometimes, notwithstanding the precaution, the stomach gets fatigued, and digestion is indolent and accompanied by nausea. This inconvenience is easily avoided by recommending the patient to take light wines with their meals, proportionate to the quantity of opium employed. Thus, suppose the quantity of opium taken daily amounts to fifteen grains or thereabouts, from a pint to a quart of wine should be allowed. Constipation, so common a consequence of taking opium, is almost certainly avoided by this means ; and an excess of sleep, is quite exceptionable. (*American Journal*, Oct., 1856.) These cases abundantly demonstrate that opium is an alterative.

In *Typhoid Fever*, opium is employed by most practioners in various forms, to meet numerous objects which each one regards it capable of filling. Dr. Graves, of Dublin, treated his patients with much regularity and a similar uniformity with hydrarg cum creta, and Dovers' powder. Success flattered the course. But believing that mercurials exert little or no necessary control over typhoid action, to lessen its grade, or shorten its course, and believing also that the beneficial portion of Graves' treatment was due to the un-accredited opiate, I have for several years in my practice, abandoned the one, and centered my confiding trust upon the other. Dovers' powder or morphia, and brandy, therefore constitute the principal medication of some twenty-five cases of this disease; two of which proved fatal. Hence it is more than probable, if practitioners would choose to let their patients lean upon it in typhoid fever, it would take the place of many other remedies, and repay its selection.

In inflammation of the neck of the uterus, which is essentially a local disease, and frequently of a chronic character, opium, in its treatment, is competent to play a conspicuous part. In aggravated cases that have advanced to ulcerative conditions, local escharotics are among the first remedies, and opium is the second. But many of them present a mild form, and exist short of erosion, when local caustics are useless, or at least unnecessary, then opium comes in as the strong hold to do the work of an unquestioned remedy.

Several of these mild cases I have treated almost exclusively with this drug, and sometimes adding an occasional blister. Opium not only lays a gentle hand upon painful vigils, but I cannot avoid the conviction that it acts as a revulsive upon the uterine circulation, as external cold is believed to act upon the cerebral circulation. It was upon this ground that we entered our opinion that opium was adverse to a full vein of venereal orgasm.

3. *The powers of Opium in Inflammatory and Febrile Diseases.*—From March till May or June in this latitude, there are frequent catarrhal affections of the throat and air passages to be met with and treated.

Some of these attacks introduce phthisis, pneumonia, obdurate bronchial disease, or inflammation of tonsils and fauces; the latter of which if not at once abridged, may continue from six to thirty days. Take then that group of symptoms which generally represent the generic outline of each. The patient, from exposure, fatigue, or atmospheric vicissitude, has had a chill; he is stuffed up, breathes with an embarrassed or wheezing respiration; has a sense of tightness across the chest, and tendency to cough; or perhaps, he has a red and painful sore throat. He may have head-ache, eyes red and watery, schneiderian membrane thickened, and discharges an augmented excretion; in other words, the patient declares he "has taken a violent cold."

Now to mend this state of things without delay, a large dose of camphorated Dover's powder—ten to fifteen grains—(of which one quarter is opium) taken at night for three successive nights, will blench nearly every case. They are at once relieved, and in from three to six days they are as well as ever. They have no need of purgatives, emetics, antimony, mercury, or other alternatives. It is proper to say, the febrile action of many of these cases will be more happily met by preceding the opium with veratrum.

A course of treatment identical with this was pursued by Dr. Henry, of Pekin, Ill., many years ago. To his original mind, bold hand, and athletic judgment, the profession is made a debtor for a certain amount of knowledge of the powers of opium, hitherto unknown. Dr. Henry was the first who presumed to treat intermittents with this agent as the chief remedy. Until he published the results of his observation, no practitioner dared to give but a grain or two of opium at a dose; and when he announced the economy of giving 4, 6, 8, and 10 grain doses, the medical faculty were ready to scream with terror at his temerity. His practice which stood out with such a touch of boldness, was characterized by as remarkable a swiftness of success. His patients recovered with an unprecedented celerity. He not only demonstrated the folly of giving mercury, and the fallacy of its action in intermittent and bilious fevers, but he made a sound reduction in the amount of quinine employed. Yet at the time these observations were published, he continued to give calomel with opium in these fevers, as "a convenient purgative." Confronting the old idea of waiting till the paroxysm of chill and fever had passed, he lost no time in administering relief, he gave his remedy as soon as he arrived to the bed-side of his patient.

We quote one case of "Congestive Intermittent" as an illustrative sample: I was called to Mrs. S. She had been taken the evening before with what she supposed to be a simple chill and fever. The chill had lasted some six or eight hours, accompanied as she expressed it, with a burning fever, with a feeling of freezing all the time, with the sensation of a loaded wagon runing over her breast. She was lying in bed comfortable, and free from fever, as she supposed; but expressed herself alarmed for the consequence of the next chill, which was expected in the course of two hours. On feeling her pulse, I readily detected that peculiar, but indescribable pulse, always found during the intermission in this form of disease. She said she felt tired, and when she attempted to sit up, became sick at her stomach. I was satisfied that the next paroxysm, from the general appearance, would prove fatal, unless prevented, or greatly mitigated, and there was no time to be lost. Accordingly, I administered in a little coffee, five grains of quinine with five of opium, and left five more of quinine and two of opium, to be given in six hours, and directed hot teas: but before leaving the house she complained of feeling cold, and commenced breathing quick and labor ously. On going to the bed-side, I found her hands cold; pulse small and rapid. The powder had only been swallowed fifteen minutes, not long enough to produce any sensible effect on the system, still I had every confidence it would moderate the paroxysm. I applied a strong mustard poultice to the stomach, which acted promptly, improved the breathing, and by this time the powder began to act. The pulse became slower, and more distinct, drops of sweat showed themselves upon the forehead, and in one hour from the time the dose was administered, she was perspiring freely, and said she felt better than she had for two days before; and being satisfied she was safe, I retired for the night, repeating the direction to have the second powder taken at the end of six hours. The next morning I found her entirely comfortable; had not slept much during the night, but felt disposed to sleep then. I directed a dose of oil, which operated in two hours very freely, when I repeated the quinine in five grain doses, without opium, and left my patient convalescent. The next day she was up and doing her work.—*Ill. and Ind. Med. and Sur. Journal, Dec.*, 1847.

Whoever has studied intermittent fever, or has experienced in his own person an attack, knows it is, as McIntosh says of Rheumatism, "a disease very unpleasant to bear." It musters an army of evil sensations, and each sensation is armed with a pointed spear of pain. Opium will not only vanquish every belligerent pang, but it well nigh clears the field of miasmatic marauders. It abates the order of march of this morbid action. These powers are exerted, says Dr. Henry, through the sedative action of the drug, given in four to eight grain doses, every six to eight hours.

A cautious consideration should steady the hand when opium is exhibited to infants. Dr. Thomas D. Mitchell, in his Materia Medica and Therapeutics, published in 1850, employs this singular language. He says: "But I desire to express my opinion in reference to the administration of opiates to infants; and I mean to do so emphatically. I take the position that an infant under a year old, never requires the medicine internally if at all. It should never be given to such tender subjects on any pretext whatever; first, because it is not necessary to their health or comfort, and second, it is truly a poison to their tender tissues."

This much we quote only to take a mammoth exception to it, and to place his injunction at the north pole of our medical esteem. Every practitioner must feel, at the perusal of this detracting sentiment, that it puts to torture countless facts of almost daily occurrence. Dr. Mitchell must know that infants are often sick, as well as adults. That like adults they suffer pain, and their claim upon medical science for speedy relief is equally imperative. Now, to deny a child the most grateful means of relief in our hands to give, because it is little, and unable to defend itself, is a trangression of a rule of humanity, pays a poor compliment to our profession, and demands it to pause, and make here a beggarly stand. If an infant is tender and susceptible, it is therefore more susceptible to medicines, a responsiveness which holds to the advantage of the discreet physician. It then requires a less dose. Shall homœopathy, which is puffed with a monopoly of doses intensely divided, be furnished with a driveling argument, by reason of this repulsive mode of standing back? Can a child so small and tender, that even a giant remedy may not be reduced in size, exactly fitted to the infantile state? No! We answer in the austerity of scientific confidence, no! If there be a microscopic disease, why not be rational, and give a corresponding microscopic remedy? If there are nice points to weigh while prescribing for infants, though it be the thousandth or ten thousandth part of a grain, is it not the business of the skilled physician to calculate them? Any dolt can prescribe a coarse dose for a plain case, but refined distinctions, based often upon narrow postulates, characterize the labors of the scientific physician.

Dr. West, one of the highest authorities on diseases of children, hesitates not to give opiates to infants under the same indications which necessitates their use in the adult. Infants from a few days to a few months old, are frequently liable to sudden fits of colic. The little sufferers will cry and writhe under it most piteously. Sage tea, a little brandy, or other carminative, will relieve these cases at first, but after several exhibitions they become inert and worthless. The child then suffers on, unless the case be laid under the mastery of an infallible opiate. Contrary to Dr. Mitchell's assertion, that "they never require this medicine either for health or comfort," the truth is painfully summed up in the innocent creature's bitter cries, which if not relieved, will cry for hours, or for a whole night. None will say, but that in this state it is a long way removed from a state of comfort, and a remedy is demanded. Opium will as certainly bring relief as that it is given. Here, then, by the failure of other means, the nature of the case decides in selecting this drug.

Out of a hundred cases that might be given, let me detail one, of a kind not the most common. Some two years ago, I found myself, by accident, in company with an infant eight or ten months old, which was irritable, peevish, and cried to excess. Every trifling displeasure or cross was a large provocation to cry. It would cry at night to the great detriment of its mother's repose. The child was thin and pale, but had no other appreciable error of health. Believing it was unwell, and the crying a symptom of its distress, I proposed to repair its fretful disposition by the agency of medicine. Six doses of morphia were left, one to be taken every night or two, as the case required. It was about two weeks in consuming the remedy. It improved from the ingestion of the first dose; and then got better, got fat and good natured. The mother afterwards expressed her regret that she did not obtain the medicine at an earlier period.

Dr. Mitchell says, secondly, (of opium in infancy,) "it is truly a poison to their tender tissues." To entitle this remark to acceptance, it should be accompanied with a reasonable sum of proof. It is equally dictatorial with the preceding one already discussed; similarly extravagant, and betrays a more startling disregard of the present state of toxicological knowledge. If Christison and other writers on poisons are to be believed, opium is *not a tissue poison*. Arsenic, corrosive sublimate, and the mineral acids, are, to use his expression, tissue poisons; they destroy the integrity of the texture of a part; while opium, strychnia, and hydrocyanic acid, are cerebro-spinants, or poisons which abolish the functions of an organ, but they meddle not with the mechanism of the tissues. "In poisoning by acrid substances," says Pereira, "opium is used with advantage to lessen the susceptibility of the alimentary canal, and thereby diminish the violence of these local irritants." There is palliation for such hap-hazard usage of language as that employed by Dr. Mitchell in the inexperienced extempore speaker, but to find it in the text of the undisturbed deliberations of a writer, deters forgiveness, and entitles them to the disdain of ascetic censure.

A convenient and safe rule for the administration of opium to infants under a year old, is to give a drop of officinal laudanum for every month in the child's age. But elixir paragoric is the better, and the generally sufficient preparation for the first few weeks of the child's existence, should an opiate be needed.

The large doses of opium employed by Dr. Henry, did not bring with them the corresponding increase of evil effect of the drug. The consequent constipation of the bowels and nausea were in no degree augmented above those effects from ordinary doses. While a state of morbid action furnishes an occasion, or a reason, why such large portions are borne with advantage, yet it does not follow that they would prove poisonous in a state of health, excepting in extremely rare cases, wherein there is an idiosyncracy against it. For adults the poisonous dose does not appear to be reached much short of twenty grains. A few may be poisoned with a less dose, but many will escape the toxic influence of a much larger quantity. "A woman desired to get rid of a useless husband laboring under a disease regarded as incurable. She gave him twenty grains of opium at a dose, yet was most grievously disappointed. A drenching perspiration came on, followed by a profuse urinary evacution, and he was cured."

The *Annals de Hygiene*, for Jan., for 1845, furnish a more remarkable case. "An apothecary's assistant, aged twenty-four years, swallowed at noon fifty-five grains of acetate of morphia in two ounces of gum water. He then sent a letter to his master, informing him what he had done. The master came half an hour after, and administered, with difficulty, and without effect, two grains of tartar emetic, followed by an ounce or two of sweet oil. Up to this time the chief symptoms were slight giddiness, which soon vanished, and the lad went out with some companions and drank half a bottle of beer. At the end of an hour and a half he complained of more giddiness, a peculiar feeling of the limbs, and a tendency to sleep. At the end of two hours he was taken to the hospital, put to bed, and made to take three grains of tartar emetic and twenty-four of ipecacuanha, in two doses, with an interval of half an hour. Vomiting could not be affected without the aid of an irritant to tickle the throat, and then it was too slight to do good.

The aspect of the case grew more alarming, till at length he fell into a profound sleep, and seemed like a drunken man. During all this the breathing continued quite natural. A pound of blood was taken from the arm, soon after which he rallied a little, and complained of a difficulty of swallowing. The pulse, which up to this time, was soft and frequent, became more full, hard and slow, and there was troublesome itching of the forehead, nose and lips. Various external irritants, and agitation of the whole body were resorted to in vain. The face was distorted, the eyes dull and sunken, the entire surface of the body of an icy coldness. At the end of three hours iodine and iodide of potash were administered, and vomiting ensued. Strong coffee frequently given, had a like effect. At the end of five hours, a third bleeding was performed, strong coffee pretty freely taken, and two blisters applied to the chest. From this time he began to improve, and converse a little. At the end of six hours he inquired whether he was not very ill, and wondered that he had survived the monstrous dose of fifty-five grains of acetate of morphia. In two or three days he was well."

The following recent case is worthy of note, and of quotation: Dr. Hays related to the college the particulars of a case in which a child, not quite six years old, was given a powder containing seven and a half grains of opium, with the same quantity of prepared chalk, the former having been substituted by mistake for rhubarb. Dr. H. did not see the patient until fourteen hours after the powder had been administered. He was told that the child, after taking the medicine seemed much excited; this was followed by restlessness and drowsiness, which continued at the time of Dr. H's visit. No vomiting had taken place. The narcotism was at no time very profound. It gradually wore off, and at the end of three days had entirely disappeared.—*Amer. Jour. Med. Sci., April*, 1859.

In comparing the powers of opium with morphine, they are usually stated to stand as 1 to 4, or that a grain of morphia has four times the strength of one grain of opium. I am led to question this position. In the absence of positive data by which to determine, I can only offer an opinion. Aside from this rule, if asked my judgment, drawn from daily observation of the two articles, I should say they stood as 1 to 2. This view is sustained by the statement of the opium eater, mentioned in the foregoing pages. He says: "I always reached the full amount which as a habit I ever used, that is either half an ounce of opium, or a quarter of an ounce of morphine per week."

Before leaving the posolegy of our subject, it is in order to discuss the incompatibility of opium with other drugs.

*Opium and quinine* are many times in western practice, mingled together in a case, and in the same prescription, or even in the same dose; and that, too, without any fear of perverting antagonism. Recently, however, an article in an Eastern journal, states what the writer regards as an incompatibility between these agents. Looking into the phenomenon of natural sleep, he found the cerebral vessels always in a state of congestion; that opium induces sleep by bringing about this same state of congested cerebral vessels. Opium, then, induces sleep by an action which is but the imitation of nature—a natural sleep by artificial means. Quinine, on the other hand, induces the opposite state of the cerebral vessels. A plethora of them, according to this writer, is removed. It therefore, like coffee, diminishes the tendency to sleep. Admitting the statement to establish this incongruity of operation, that quinine does arrest the soporific effect of opium, the fact is void of objection, though they be given in conjunction for the purposes of anodyne, alternative, or sedative effects.

Question? In view of this conflicting action, may we not glean from it a useful gem of practice? Instead of its being wholly an obtrusive feature in the premises, may it not prove of eminent service? Why will not the addition of quinine to an opiate, diminish the dangers of that opiate, when given to infants, lessen our trepidation of employing it, and thus extend its usefulness in that class of subjects, by antidoting one of the dreaded properties of the drug, viz: the tendency to stupor.

Among the recent movements in the progress of medical practice, the heroic plan of administering opium in *puerperal fever* is the most decisive. Dr. Barker, of New York, speaks of it in terms of high commendation. He regards this fever as differing from most other inflammations—as being a zymotic disease, and which bears a kindred identity with erysipelas. That, like erysipelas, it often springs from contagion, and that prophylactic measures secure to the patient much safety, which peculiarity widely separates it from a simple inflammation. He does not look upon opium as *the* remedy in this disease, nor does he employ it because the patient has puerperal fever, but because there is in this terrible plague a clear indication for its use, which indication is rarely absent. He says: An important indication in the treatment of this disease is to control vascular excitement and nervous irritation. As an arterial sedative, well adapted to reduce the excitement of the vascular system, inasmuch as it does not depress the vital powers, Dr. B. recommends the veratrum viride. To allay nervous irritation he is in favor of large and repeated doses of opium.

It is astonishing to see to what an extent patients will tolerate opium, where the peritoneal lesion predominates. When opium is acting benficially in such cases, there will be a reduction of the frequency of the respiration, and also of the pulse. If opium is pushed to the point of incipient narcotism, and the respirations grow slower without a corresponding decrease of the pulse, the remedy is not acting beneficially, and should at once be abandoned. Illustrative of an occasional necessity there is to reduce the pulse, while you at the same time continue the effect of the opiate, he relates the following: "The patient had been freely impressed with opium, which had reduced the number of respirations to a low standard, while the pulse continued elevated to the pitch of 120 or 130. This disproportion in two vital movements began to indicate a cyanosis of blood, and depict a livid countenance. In place of the opiate, veratrum was given which lessened the cardiac to a normal correspondence with the respiratory movement. The dusky features disappeared, and the patient recovered."

In the decline of an epidemic in England, Dr. Ferguson began to rely upon ten grain doses of Dover's powder in the outset of this disease, or whenever a parturient female began to complain of a pain in the abdomen. It removed the suffering, and with a few exceptions, arrested the disease. Permit the report of a single case observed lately. Mrs. K., two months advanced in gestation, had free uterine hemorrhage for a day or two, from supposed mechanical injury of the organ. On the mornings of Tuesday, Wednesday and Thursday, she had vigorous chills, followed each day with a smart fever. Visiting her Thursday at 3 P. M., she was found with an ardent heat of skin, thirst, nausea, and vomiting, painful and tender abdomen, head-ache, and a pulse of 150 per minute. Twenty grains of camphorated Dover's powder, containing about six grains of opium, were immediately given. She continued to retch in efforts to vomit for three hours, when three doses of veratrum were given, hourly, with relief to the stomach. The next morning the patient was found sitting up, free from fever, free from every distress, and from tenderness of the bowels, with a pulse beating at 100. A few more doses of veratrum and opium completed her recovery.

Regarding the publication of Dr. Henry's views, as adding the property of affluence to our knowledge of opium, and feeling a faltering perplexity with the usual bootless, and even contradictory lines of treatment laid down for *dysentery*, I began eight years ago to employ opium in this disease.

From its diligent efficiency sprung a ripening satisfaction, which gave a proneness to rely upon it, with only such varying interruptions as special indications might require. Generally five or six grain doses of this drug, in the form of camphorated Dover's powder—the camphor being substituted in place of the ipecacuanha—were given in the outset every night, or every night and morning. This at once subdued many and many a case which, under other plans of treatment must have painfully continued for many days, or many weeks. Twenty-four hours to four days, is the ordinary range of duration of dysentery thus treated. Whereas hundreds of accomplished practitioners will testify to a frequent and troublesome duration of two to four weeks, under the usual methods of treatment.

Neither Dr. Henry nor any other writer within my knowledge, had led the way, or authorized us to address dysentery solely by the ruling powers of opium. But though without the prestige of precedent, the course was legitimately deducible from known truths, and from Dr. Henry's premises. Favoring results alone encouraged my course of managing this disease, until July, 1857, the *Amer. Journal Med. Sciences* praised it in authoritative terms. After reviewing Dr. Tiedimann's plan of treating dysentery, who proposed twenty grain doses of nitrate of potassium, given three or four times a day; the reviewer says: "We cannot agree with Dr. T. in his denunciation of opium as positively mischievous in the early stages of dysentery. We are in the constant habit of giving it from the very onset of the disease, and always with the very best effect. To derive from it the good it is calculated to produce in this disease, it must, however, be given in large doses. The effect of small and repeated doses is rather mischievous than beneficial. Although we have generally found sporadic dysentery a troublesome and obstinate, rather than a fatal disease, we certainly have not been quite so successful in its treatment as Dr. T. In few cases, occurring in very young, or in diseased or broken down constitutions, the disease has terminated fatally. We very much doubt whether in these cases the termination would have been different had we subjected them to the treatment laid down in the essay of Dr. Tiedimann.

In pledging the far-reaching œsculapian hand of opium, to save both time and life, within a reasonable certainty, in this malady, we do not lose sight of the kind of cases to be met with. It is not forgotten that dysentery, like croup and rheumatism, presents two different grades. The one associated with febrile or inflammatory features, the other being without fever. But instead of letting the distinction likewise divide our treatment, we follow the plan adopted by Dr. Jenner Coxe, of New Orleans, respecting the treatment of croup, namely, group the different varieties in one, and apply the same remedial measures, only with such variations as the special aspect of each case may demand. In a mere, yet imperative, action of the bowels, with tenesmus, scanty, and muco-sanguineous evacuations, one or two full doses of opium will command relief. Add to this derangement a fever, thirst, heat of abdomen, and an antecedent chill, and you have the other variety of dysentery; which is still answerable to a larger number, and larger sized doses of the agent. Veratrum viride and local fomentations will add to our resources, but it is rare that recovery is a contingent of them.

Nor is it to be supposed that opium can be confided in when the diseased action has gained that destructive advance known as the ulcerative stage. Even here it cannot be prudently dispensed with, but its value is subordinate to other remedies. While it will quiet the vermicular action of the bowels, lessen the determination of blood to the pelvic viscera, sooth the wincing tenesmus kept up by the rough surface of eroded texture; local appliances, such as injections of nitrate of silver, and its kindred, must take priority. Neither in epidemic dysentery can the favorable results of opium be promised with as much hope, or trusted in, so exclusively. But if called in season to a sporadic case of dysentery, the alpha of the curative plan is, not a purgative, nor an emetic, nor calomel, but an arbitrary portion of opium, from the extrotic effect of which, there is plethoric reason to believe, the mischief will be arrested, and the subsequent stages of the malady will be anticipated.

Thus we have written of this herculean therapium. We have contemplated the principal spheres embraced in its mission and interrogated the scope of its powers. Our studies have evolved, in the foregoing essay, now, and not yet admitted points which may be in numerical order, re-stated as follows:

1. Contrary to the authority of Pareira, opium allays both normal and abnormal salacity.

2. It delectably exilerates the mental faculties of only about one half who take it.

3. Of opium eaters and those requiring to resort to it; it does not always require of them to increase the amount, in order to obtain the same result.

4. Those habituated to the use of tobacco, are generally freed from the secondary effect of nausea.

5. The relative power of morphia to opium, by weight, is believed to stand as 1 to 2; instead of as 1 to 4. Which is, that two grains of opium equals one grain of morphine.

6. Opium and quinine are in some respects incompatible with each other, which property may be usefully employed as antagonistic to stupor when prescribed for infants.

7. Without becoming intoxicated with this drug, and short of being proved guilty of adulation, we say, opium stands a head and shoulders above any other remedy, or other treatment in dysentery.

